# A single dose of lipopolysaccharide elicits autofluorescence in the mouse brain

**DOI:** 10.3389/fnagi.2023.1126273

**Published:** 2023-03-20

**Authors:** Yanzhuo Yang, Qingting Yu, Bin Li, Shijia Li, Zuisu Yang, Falei Yuan, Zhongliang Liu

**Affiliations:** ^1^Department of Pharmacy, School of Food and Pharmacy, Zhejiang Ocean University, Zhoushan, China; ^2^Zhoushan Institute for Food and Drug Control, Zhoushan, China; ^3^Department of Oncology, Zhoushan Hospital of Traditional Chinese Medicine, Zhoushan, China

**Keywords:** autofluorescence, aging, neuron, lipopolysaccharide, lipofuscin

## Abstract

One hallmark of aging is autofluorescence (AF) in the brain. However, the underlying mechanism for inducing AF remains unknown. This study aims to determine the cause(s) of this phenomenon. The endogenous expression pattern of AF in mice was examined at differing ages. Intraperitoneal injection of a single dose of lipopolysaccharide (LPS) was performed to induce AF. Copper sulfate was applied to remove AF to allow for further immunofluorescence staining. AF appeared in the mouse brain as early as 3 months of age. In the cortex, AF occurs in the lysosomes of microglia, astrocytes, endothelial cells, and oligodendrocyte lineage cells and its prevalence increases with age. Interestingly, AF never occurs in the pericytes of young or aged brains. LPS administration resulted in a rapid and marked induction of brain AF, similar to the normal aging process. Finally, age-related and induced AF can be eliminated by low concentrations of copper sulfate solution. This pre-treatment is safe for aging and lineage tracing studies. These findings depict that AF in the brain could be associated with the innate immune response against Gram-negative bacteria infection.

## 1. Introduction

Lipofuscin is an age-related pigment abundant in neurons, myocytes, and skin cells. Hannover first observed it in the cytosol of aging nerve cells in 1842 (Porta, 2002). In addition to lipofuscin, elastin and porphyrins (found in red blood cells) are also capable of autofluorescing. Typically, autofluorescence (AF) occurs in postmitotic cells, especially normal aging neurons, in patients with pathological conditions, including strokes and tumors (Zhang et al., 2022). AF can also be observed in the astrocytes of damaged/traumatized brains and is a hallmark of cell senescence (Castejón, 2015). Skin AF has been utilized to examine the association between type 2 diabetes and brain atrophy, yet the pathogenesis of the AF has not been fully understood (Moran et al., 2015).

Lipofuscin is considered toxic as it is cellular waste that can be neither degraded nor ejected from the cell. Therefore, AF from lipofuscin can result from oxidative stress (Croce and Bottiroli, 2014). Lipofuscin formation appears to share the same mechanisms that cause aging (Terman and Brunk, 1998). Lipofuscin has an emission wavelength between 460 and 630 nm, with a maximum fluorescence intensity of approximately 578 nm (Moreno-García et al., 2018). It is essential to understand how and where AF develops in the brain to prevent it from interfering with staining procedures. Red fluorescent proteins (RFPs) have been widely utilized for labeling glial cells, such as astrocytes, in fate mapping. This is partly because green fluorescent proteins (GFPs) are soluble in water if not properly handled and fixed using paraformaldehyde (Jockusch et al., 2003). Animals undergoing fate mapping must be (i) genotyped and (ii) injected with tamoxifen (usually for five consecutive days, followed by a gap of at least 8 days to exclude the residual tamoxifen effects) (Valny et al., 2016; Shi et al., 2021). These laboratory animals could become sufficiently mature to develop endogenous AF. Lipofuscin fluorescence has been observed in human neurons in patients as young as 3–4 months (Porta, 2002), However, no such data is available in mice. A marked increase in AF was observed in the macrophages of mice after the intraperitoneal injection of tamoxifen suspended in the plant oil (Bulut et al., 2021). However, a dramatic decrease in AF was observed when tamoxifen was administered to the diet. Thus, the authors believed that the AF was from the plant oil and then picked by macrophages.

It is reported that 70% of mouse microglia exhibit AF as an aging function (Burns et al., 2020). Microglia actively remove myelin debris; however, this debris increases with age, leading to an increased burden on microglia to clear this cellular waste. Instead of being expelled, the debris is isolated by formatting lipofuscin-like lysosomal inclusions (Safaiyan et al., 2016). AF pathogenesis in other cell types, including astrocytes, oligodendrocytes, oligodendrocyte precursor cells (OPCs), and blood vessel cells has received minimal attention. However, one study found age-dependent AF granules in the mouse retinal blood vessel (Xu et al., 2008). In addition, the intensity of cerebral vascular AF was higher in Alzheimer’s disease (AD) patients than in age-matched controls (Christov et al., 2008). Pathogen infection, particularly by Gram-negative bacteria as one of the possible etiologies for AD, has received growing attention (Zhan et al., 2018; Lukiw et al., 2019). Lipopolysaccharide (LPS) are toxins found in the outer layer membrane of Gram-negative bacteria. They can be recognized by the transmembrane protein, toll-like receptor 4 (TLR-4), and the cytosolic receptor, cysteinyl aspartate specific proteinase-11 (caspase-11). An LPS molecule is usually comprises three components: the hydrophobic and toxic lipid A triggering the endotoxin response; a core oligosaccharide; and an O-antigen (Raetz and Whitfield, 2002). LPS originating from *Escherichia coli* has been found in the cortex of both “normal” and AD human brains. and it was localized in the peri-nuclear area of neuron-like cells (Zhan et al., 2016). Furthermore, we investigated the relationship between lipofuscin and LPS entry into the brain since lipofuscin and brain LPS can be detected by periodic acid-Schiff (PAS) staining (Benavides et al., 2002; Zhan et al., 2021).

This study determined the expression profile of AF in the brain over time. The results indicated that AF appears in the cortex, hippocampus, and hypothalamus of young mouse brains. Brain AF was elicited with a single dose of LPS, administered through intraperitoneal injection, resembling the AF from normal aging. Finally, we optimized the copper sulfate method for eliminating AF to improve the results of further immunofluorescence staining and RFP-based lineage tracing.

## 2. Materials and methods

### 2.1. Animals

Male C57BL/6 mice (4 weeks, 6 weeks, 3 months, 6 months, 12 months, and 18 months) were purchased from Hangzhou Ziyuan Inc., (Zhejiang, China) and housed at an animal facility. All animals had unlimited access to food and water. To induce AF, mice received 5 mg/kg LPS derived from *E. coli* 055: B5 (#ST1470, Beyotime, Shanghai). Animal experiments were approved by the Experimental Animal Ethics Committee of Zhejiang Ocean University (# SCXK ZHE 2019-0031).

### 2.2. Immunohistochemistry and AF detection

All mice were euthanized by using carbon dioxide and transcardially perfused with saline, followed by 4% paraformaldehyde. Brain tissues were fixed in 4% paraformaldehyde at 4°C for 1 h; embedded in low-melt agarose (k07711, KehBio Inc., Beijing) at 37°C and sectioned by a vibratome (ZQP-86, Zhisun Equipment Inc., Shanghai). Sections were washed with phosphate-buffered saline (PBS), permeated by 0.3% Triton X-100, and blocked by 1% bovine serum albumin in a 24-well cell culture plate. For immunofluorescence staining, tissue samples were incubated with primary antibodies (neuronal nuclei, NeuN, 24307, CST; cluster of differentiation 11b, CD11b, 557394, BD Bio-sciences; glial fibrillary acidic protein, GFAP, D262817, Sangon Biotech; cluster of differentiation 31, CD31, 550274, BD Biosciences; Sry-related high-mobility-group/HMG box 10, Sox10, AF2698, Beyotime; platelet-derived growth factor receptor-β, PDGFR-β, AF1042, R&D; lysosomal associated membrane protein 1, LAMP1, ab208943, Abcam; β-amyloid 42, Aβ42, GTX134510, Genetex) overnight at 4°C. After washing three times with PBS, immunofluorescence secondary antibodies (111-095-003, 705-095-147, 112-095-003 Jackson ImmunoResearch) were applied at room temperature for 1 h. Brain sections were finally imaged using immunofluorescence microscopy (Olympus BX41). For AF detection, rhodamine channel with an excitation wavelength of 450–490 nm was selected. For 3,3’-diaminobenzidine tetrahydrochloride (DAB) staining, tissues were incubated with citrate-EDTA antigen retrieval solution (Beyotime, Shanghai) at 90°C for 20 min, and then incubated with primary antibodies (LPS core, WN1222-5, Hycult Biotech; Aβ42, ab201060, Abcam) overnight at 4°C. The samples were again washed three times with PBS, after which DAB staining was performed in conjunction with a polymer horseradish peroxidase detection kit (PK10006, Proteintech) and imaged using light microscopy.

### 2.3. Western blotting

Fresh brain tissue was sonicated and lysed by radioimmunoprecipitation assay (RIPA) buffer. Protein lysate was separated by sodium dodecyl sulfate-polyacrylamide gel electrophoresis (SDS-PAGE) and then transferred to a piece of polyvinylidene difluoride (PVDF) membrane. The membrane was incubated with lipid A (ab8467, Abcam) and α-Tubulin (T9026, Sigma) antibodies overnight at 4°C and then incubated with a horseradish peroxidase-conjugated secondary antibody (115-035-003, Jackson) for 1 h at room temperature. Finally, protein bands were detected using an enhanced chemiluminescence (ECL) solution and analyzed with Alphaview SA software from Fluor Chem FC3 (ProteinSimple Inc., California, CA, USA). Western blot analysis was performed using α-Tubulin as a control.

### 2.4. Prussian blue, immunoglobulin G (IgG), and senescence β-galactosidase (SA-β-Gal) staining

Prussian blue staining (Solarbio, Beijing) and senescence β-galactosidase staining (Beyotime, Shanghai) were performed following the manufacturer’s instructions. For IgG staining, tissues were treated and stained using the same methodology described for DAB staining, except for the incubation with primary antibodies.

### 2.5. Copper sulfate bleach of AF

Brain sections from 18 months old and 14 days post LPS injection C57BL/6 mice were treated with varying concentrations of copper sulfate for 1.5 h. Sections were then performed with immunofluorescence staining. To assess the effects of copper sulfate treatment on the fluorescence of RFP in a lineage tracing system, Sox10-cre/ERT2 and tdTomato reporter mice (Jackson Laboratories, Maine, ME, USA) were cross-bred. Tamoxifen was injected into the offspring, using a dosage of 1 mg per mouse per day for 3 days. The sample brains were harvested and sectioned using the vibratome method described above.

### 2.6. Statistical analyses

Each experiment in this study was carried out in at least triplicate. All quantified data are shown as mean ± standard error (SE). Significance analysis uses one-way analyses of variance (ANOVA) or Student’s t test. A *p*-value less than 0.05 (*p* < 0.05) was considered statistically significant.

## 3. Results

### 3.1. AF of the brain is age dependent

**FIGURE 1:**
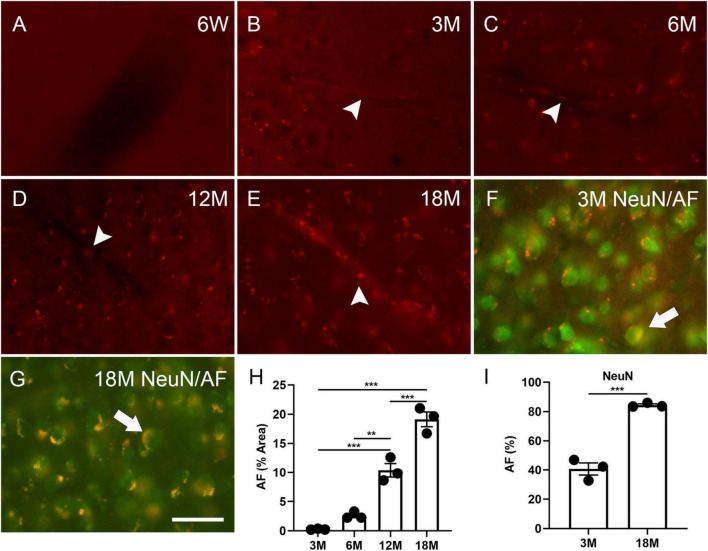
Autofluorescence (AF) in the cortex of mouse brains at specific ages. The representative images of AF at 6 weeks (6W, **A**), 3 months (3M, **B**), 6 months (6M, **C**), 12 months (12M, **D**), 18 months of age (18M, **E**). Blood vessel-like AF was found at 3 months of age and older (arrowheads, **B–E**). The representative images of AF (red) co-localized with NeuN (green) at 3 months and 18 months of age (arrows, **F,G**). Area percentage of AF at different ages and in neurons at three and 18 months of age **(H,I)**. The scale bar represents 20 μm. The data are represented as mean ± SE. Significance analysis with one-way analyses of variance (ANOVA) followed by Tukey’s multiple comparisons test or *t*-test. **p < 0.01, ***p < 0.001.

To understand the timeline of AF development, the mouse brains were examined at specific intervals (initially at 4 weeks, 6 weeks and then 3, 6, 12 and 18 months). AF was not identified in the cortex, hippocampus, or hypothalamus at 4 weeks (data not shown) and 6 weeks (Figure 1A), but was observed in these structures in brains as young as 3 months (Figure 1B and Supplementary Figure 1), which concurs with the findings of Oenzil et al. (1994) in rats. AF increased with age (Figures 1B–E, H). 0.3% ± 0.1%, 2.6% ± 0.4%, 10.4% ± 1.2%, and 19.1% ± 1.3% cells in the cortex exhibited AF at the age of 3, 6, 12, and 18 months, respectively. 40.7 ± 4.2% and 84.4 ± 0.8% of cortical neurons could express AF at three and 18 months, respectively (arrows, Figures 1F, G, I). AF was found in the blood vessel cells, indicating that neuronal lipofuscin could not be the only AF source in young and aged brains (arrowheads, Figures 1B–E).

### 3.2. AF is expressed in the lysosomes of glial and endothelial cells in young and aged brains

**FIGURE 2:**
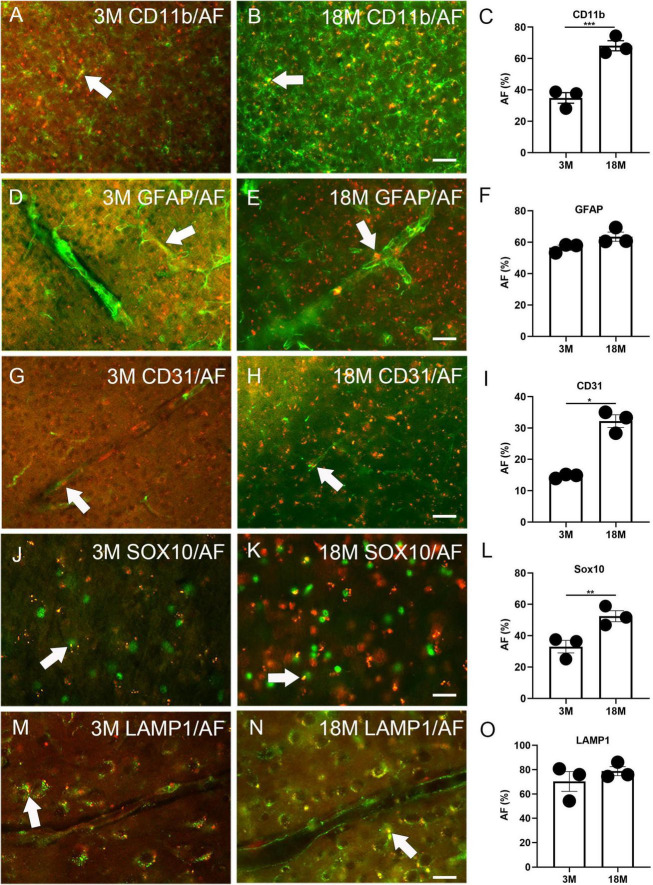
Autofluorescence (AF) expression in glial cells, blood vessels, and lysosomes of young and aged mice. The representative AF images (rhodamine channel) co-localized with CD11b **(A–C)**, GFAP **(D–F)**, CD31 **(G–I)**, Sox10 **(J–L)**, and LAMP1 **(M–O)** among young (3M) and aged brains (18M). The arrows depict fluorescence coincident with AF. The scale bars represent 40 μm **(A,B,D,E,G,H)** and 20 μm **(J,K,M,N)**. The data are represented as mean ± SE. Significance analysis with *t*-test. *p < 0.05, **p < 0.01, ***p < 0.001.

Immunofluorescence staining was performed to determine any AF occurrence in glial and blood vessel cells. Among three-month-old mice, 34.9% ± 3.4% CD11b+ microglia, 56.5% ± 1.7% GFAP+ astrocytes, 14.6% ± 0.4% CD31+ endothelial cells, and 33.0% ± 4.0% Sox10+ oligodendrocyte lineage cells were co-localized with AF (arrows, Figures 2A, C, D, F, G, I, J, L). Therefore, these cells can develop AF as early as 3 months of age. In aged brains, 68.1% ± 3.3% CD11b+ microglia, 63.5% ± 2.9% GFAP+ astrocytes, 32.2% ± 2.0% CD31+ endothelial cells, and 52.4% ± 3.5% Sox10+ oligodendrocyte lineage cells were co-localized with AF. Thus, the prevalence of AF positive microglia, endothelial cells, and oligodendrocyte lineage cells significantly increases with age. However, the propensity of astrocytes to exhibit AF did not alter (arrows, Figures 2B, C, E, F, H, I, K, L). Interestingly, PDGFR-β did not co-localize with AF in either young or aged brains, indicating that pericytes are not responsible for AF (Supplementary Figure 2). We did LAMP1 immunofluorescence staining to determine which cellular structure produces AF. It was observed that AF occurred in lysosomes, and 70.3% ± 8.1% and 78.8% ± 3.7% of lysosomes exhibited AF among young and aged brains, respectively (arrows, Figures 2M–O).

### 3.3. A single dose of LPS induced brain AF

**FIGURE 3:**
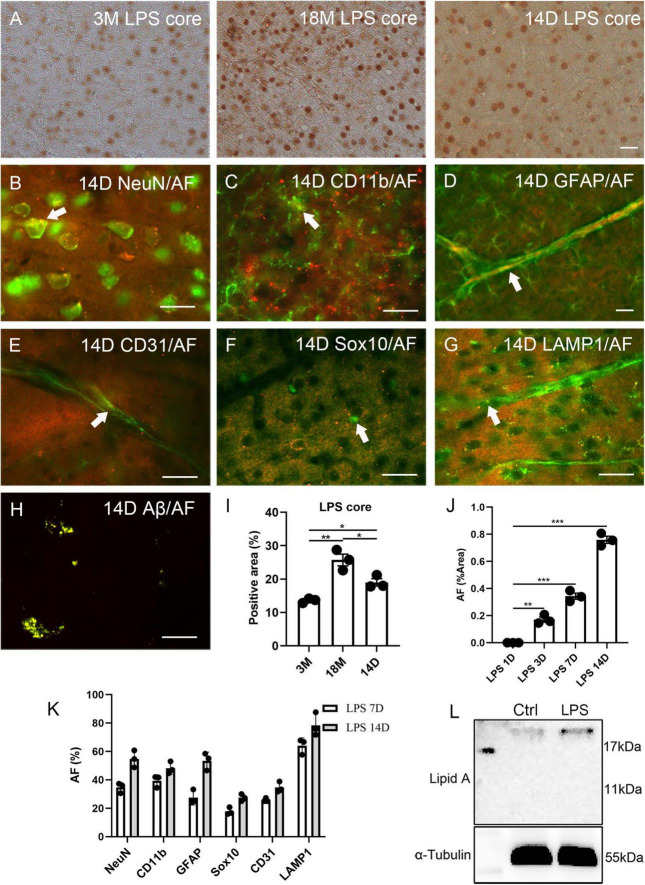
Correlation between lipopolysaccharide (LPS) and AF within mouse brains. The representative images of LPS core staining of mouse brains at 3 months (3M) and 18 months (18M) of age and 14 days (14D) after LPS injection **(A)**. LPS-induced AF (rhodamine channel) co-localized with NeuN **(B)**, CD11b **(C)**, GFAP **(D)**, CD31 **(E)**, Sox10 **(F)**, and LAMP1 **(G)**. LPS-induced Aβ co-localized with AF (rhodamine channel) **(H)**. The positive area percentage of LPS core at 3 months (3M), 18 months (18M) of age, and 14 days after LPS injection **(I)**. The area percentage of AF in 1 day (1D), 3 days (3D), 7 days (7D), and 14 days (14D) LPS-injected mouse brain sections **(J)**. The percentage of AF-positive cells 7 days (7D) and 14 days (14D) mice after LPS injection **(K)**. Western blot of lipid A of the cortex of 6 weeks old mice after LPS injection for 1 day and age-matched control **(L)**. The arrows indicate fluorescence coincident with AF. The scale bars represent 20 μm. The data are presented as the mean ± SE. Significance analysis with one-way analyses of variance (ANOVA) followed by Tukey’s multiple comparisons test. *p < 0.5, **p < 0.01, ***p < 0.001.

Lipofuscin is a substance positive for PAS staining. Recently, the PAS method has been used for staining the polysaccharides of LPS (Zhan et al., 2021). Therefore, we stained the brain tissue of normal C57BL6 mice for lipid A and LPS core (polysaccharide component). Lipid A was not observed in the cortex. Surprisingly, neuron-like cells were positive for LPS core at 3 months (13.6% ± 0.4% of the positive area), and the signal of the LPS core staining significantly increased with age (25.7% ± 1.8% of the positive area at 18 months, Figures 3A, I). At 4 weeks of age, the male C57BL6 mice received a single dose of LPS (5 mg/kg). The signal of the LPS core staining significantly increased 14 days after LPS injection (19.0% ± 1.1% of the positive area, Figures 3A, I), compared to the staining at 3 months. AF induction could be seen in the cortex, hippocampus, and hypothalamus at 3, 7, and 14 days after injection. Meanwhile, AF was not observed in the control group at any point or in the injection group one day after injection (Figure 3J and Supplementary Figure 3). AF induction was found in 34.6% ± 2.0% NeuN, 39.4% ± 2.5% CD11b, 27.4% ± 3.0% GFAP, 25.8% ± 0.8% CD31, 17.8% ± 1.5% Sox10 positive neurons, glial cells, and endothelial cells 7 days after LPS injection (Figure 3K and Supplementary Figures 4A–E). In contrast, it was found in 54.8% ± 3.5% NeuN, 48.3% ± 2.5% CD11b, 53.4% ± 3.5% GFAP, 34.7% ± 2.2% CD31, and 27.4% ± 1.5% Sox10 positive neurons, glial cells, and endothelial cells 14 days after LPS injection (Figures 3B–F, K). The induced AF was co-localized with lysosomes, as with endogenous AF (Figures 3G, K and Supplementary Figure 4F).

The Western blot of lipid A revealed that the LPS entered the cortical parenchyma 1 day after LPS injection (Figure 3L). Lipofuscin usually contains Aβ (Giaccone et al., 2011); thus, we examined this LPS induction model with Aβ42 staining. In the 14 days of LPS injected mice, Aβ42 was significantly over-expressed than aged mice (Supplementary Figures 5F, G). Further Aβ42 fluorescence indicated that induced Aβ42 co-localized with AF (Figure 3H). However, we could not detect SA-β-Gal in young, LPS-induced animals (Supplementary Figures 5A, C), even though Aβ42 has been reported to increase SA-β-Gal *in vitro* (He et al., 2013). However, we observed this phenomenon in aged mice (Supplementary Figure 5B). Prussian blue staining and blood IgG staining were performed to exclude the possibility of LPS-induced artifacts including bleeding or blood clots (Sumbria et al., 2016). A single dose injection of LPS did not increase bleeding, blood clots, or vascular leakage (Supplementary Figures 5D, E).

### 3.4. Removal of AF by copper sulfate pre-treatment did not affect fluorescence labeling

**FIGURE 4:**
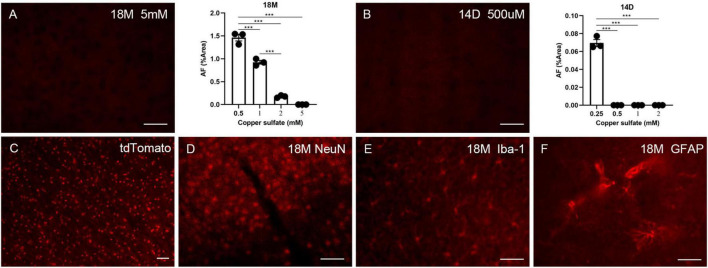
The effects of copper sulfate pre-treatment on brain AF. Effect of different concentrations of copper sulfate on the amount of AF elimination in the aged brain (18M), and representative image of complete elimination of AF at 5 mM **(A)**. Effect of different concentrations of copper sulfate on the amount of AF elimination in the 14 days of injection in lipopolysaccharide (LPS)-induced young brains (14D), and representative image of complete elimination of AF as low as 500 μM **(B)**. Red fluorescent protein (RFP) tdTomato was not affected by copper sulfate treatment **(C)**. Brain sections from aged mice (18M) were pre-treated using 5 mM copper sulfate for 1.5 h, and immunofluorescence staining was performed for detecting NeuN **(D)**, Iba-1 **(E)**, and GFAP **(F)**. The scale bars represent 20 μm. Significance analysis with one-way analyses of variance (ANOVA) followed by Tukey’s multiple comparisons test. ***p < 0.001.

Autofluorescence (AF) cannot be eliminated with solvents such as ethanol, isopropanol, or xylene, excluding the possibility that lipids solely induce AF. Other research indicates that AF can be removed using a 10 mM copper sulfate solution (Schnell et al., 1999; Potter et al., 2012). However, the AF-reducing effect of copper sulfate at significantly high concentrations is an unacceptable methodology for experiments requiring additional immune labeling. We attempted to optimize this process by increasing the copper sulfate incubation period but decreasing its concentration. Since aged brains exhibit higher AF levels, 500 μM, 1 mM, 2 mM, and 5 mM copper sulfate solutions were evaluated for eliminating AF in aged brains (18 months of age) with an incubation period of 1.5 h. Only 5 mM copper sulfate solution completely removed AF (Figure 4A). To remove LPS-induced AF, 250 μM, 500 μM, 1 mM, and 2 mM copper sulfate solutions were examined for eliminating the AF in the mouse brains that had received LPS for 14 days. 500 μM copper sulfate solution completely removed AF (Figure 4B). Then, we investigated whether pre-treatment using copper sulfate affects fluorescence labeling. Brain sections from Sox10-cre/ERT2/floxed tdTomato mice were treated using a 5 mM copper sulfate solution for 1.5 h. Fluorescence bleaching of tdTomato did not occur, depicting that copper sulfate treatment is safe for RFP labeling (Figure 4C). Additionally, brain sections from aged mice were pre-treated using a 5 mM copper sulfate solution for 1.5 h. Additional immunofluorescence staining was still available to detect NeuN, Iba-1, and GFAP (Figures 4D–F).

## 4. Discussion

This study observed that AF appears as early as 3 months of age in the mouse brain and prevalence increases with age. AF is expressed in neurons, glia, and endothelial cells except for pericytes. By injecting a single dose of LPS, we demonstrated that AF was associated with systemic inflammation. The procedure for eliminating AF was optimized for further immunofluorescence labeling.

Over fixation could also bring AF (Pfeiffer et al., 2021), but the AF of fixative origin seems unlikely since the tissues were sectioned right after 1 h fixation using paraformaldehyde. IgG DAB staining demonstrated that a single dose of LPS derived from *E. coli* did not result in vascular leakage. In contrast, mice treated with a 1 mg/kg dose of LPS derived from *Salmonella typhimurium* showed cerebral micro-hemorrhages (Sumbria et al., 2016). This discrepancy could be attributed to the type of LPS used. A similar study established that mice receiving a single dose of 2 mg/kg LPS from *E. coli* showed no IgG leakage from brain vasculature or neurodegeneration (Bowyer et al., 2020).

The early AF emergence described that secondary antibodies for the fluorescein isothiocyanate (FITC) channel seem more suitable when conducting immunofluorescence labeling. Although Sudan black B reduces AF, this treatment is not appropriate for immunofluorescence detection as Sudan black B masks fluorescence (data not represented).

The early occurrence of AF could explain for some controversial phenomena, including astrocyte-to-neuron conversion (Wang et al., 2021), OPC to astrocyte/neuron conversion (Akay et al., 2021), and endothelial-to-mesenchymal transition (Hong et al., 2018). RFP was frequently used in these studies for fate mapping. However, AF can appear in all these cells after genotyping and tamoxifen injection.

Neural/glial antigen 2 (NG2) glia involves OPCs and pericytes since the proteoglycan NG2 is also a receptor for PDGF (Attwell et al., 2016). Therefore, Sox10 was used in this study to differentiate OPCs from pericytes. This is because Sox10 is a more specific biomarker for oligodendrocyte lineage consequently, it is suitable for labeling OPCs, myelinating oligodendrocytes, and newly formed oligodendrocytes, compared to less specific markers, such as oligodendrocyte transcription factor 2 (Olig2), platelet-derived growth factor receptor-α (PDGFR-α), and NG2 (Zhang et al., 2014). Pericytes appear to renew themselves slowly because the lineage tracing system revealed that some unknown precursor cells occasionally became pericytes in normal aging mice (Kang et al., 2010). These pericytes may renew themselves faster under systemic inflammation and therefore demonstrate no detectable AF.

Lipopolysaccharide (LPS)-challenged mice had many macrophages near the blood vessels, which is one of the characteristics of the normal aging brain (Allen et al., 2023). The hyaline substance seen in the blood vessels of an aged chimpanzee could be autofluorescent intracellular lipofuscin (Gilissen et al., 2016). AF could relate to palmitoyl-protein thioesterases (PPT) in the lysosome. Ppt1 mutation can cause neuronal ceroid lipofuscinosis (NCL), a neurodegenerative disease (Gupta et al., 2001). Interestingly, neuroinflammation showed up in the Ppt1 mutant mice produced by Jalanko et al. (2005) at 3 months of age, which was before the occurrence of neurodegeneration. Environmental bacteria can enter the blood circulation through the lungs, skin, gut, and urinary tracts. Four types of Gram-negative bacteria, *E. coli*, *Pseudomonas aeruginosa*, *Klebsiella pneumoniae*, and *Acinetobacter baumannii* are responsible for up to 62% of bloodstream infections (Holmes et al., 2021). In addition, Gram-negative *E. coli* has surpassed Gram- positive *Staphylococcus aureus* as the most prevalent bacteria in the blood. It is partly due to the overuse of antibiotics like penicillin. The mechanism by which LPS gains access to the brain is unclear. Western blot cannot detect free LPS with a molecular weight of 10 kDa in this study due to the detection limit or LPS binding with other proteins (Vargas-Caraveo et al., 2017). Generally, LPS-binding protein and cluster of differentiation 14 (CD14) can help LPS bind TLR-4 and elicit innate immune responses (Ryu et al., 2017). However, high mobility group box-1 protein (HMGB1) and galactin-3 can also combine with LPS. Unfortunately, the underlying mechanism for delivering LPS by HMGB1 and galactin-3 is poorly understood (Thiéblemont and Wright, 1997; Deng et al., 2018; Lo et al., 2021). Recently, a novel, TLR-4-independent role of CD14 for cytosolic LPS sensing has been discovered (Vasudevan et al., 2022). The presence of LPS in the brain cortex had never been proved, despite numerous attempts using fluorescence or radiolabeled LPS (Li and Blatteis, 2004; Banks and Robinson, 2010). However, whether the LPS core is located in the peri-nucleus area of cortical neurons can be determined.

A previous study has demonstrated that LPS increases Aβ in transgenic mice with the Swedish mutation (K595N/M596L) of amyloid precursor protein (APPswe) (Sheng et al., 2003). However, LPS also increases Aβ42 in normal young adult mice. The virus injection potential to induce AF was not evaluated in this study. However, lipofuscin may be a part of the innate immune defense system and that viral infection may induce AF (Lemelle et al., 2020). Future studies, we will seek to determine the correlation between viruses, AF, and neurodegeneration.

## Data availability statement

The original contributions presented in this study are included in the article/Supplementary material, further inquiries can be directed to the corresponding author.

## Ethics statement

The animal study was reviewed and approved by the Animal Ethics Committee of Zhejiang Ocean University (# SCXK ZHE 2019-0031).

## Author contributions

QY and FY contributed to conception, design of the study, and wrote the first draft of the manuscript. YY and QY organized the database. YY, BL, and QY performed the statistical analysis. ZL, ZY, FY, and SL wrote sections of the manuscript. All authors contributed to manuscript revision, read, and approved the submitted version.
