# Supplementary figures and images for: A single dose of lipopolysaccharide elicits autofluorescence in the mouse brain
Source: Front Aging Neurosci. 2023 Mar 20;15:1126273. doi: 10.3389/fnagi.2023.1126273 (PMC10067636; doi:10.3389/fnagi.2023.1126273)

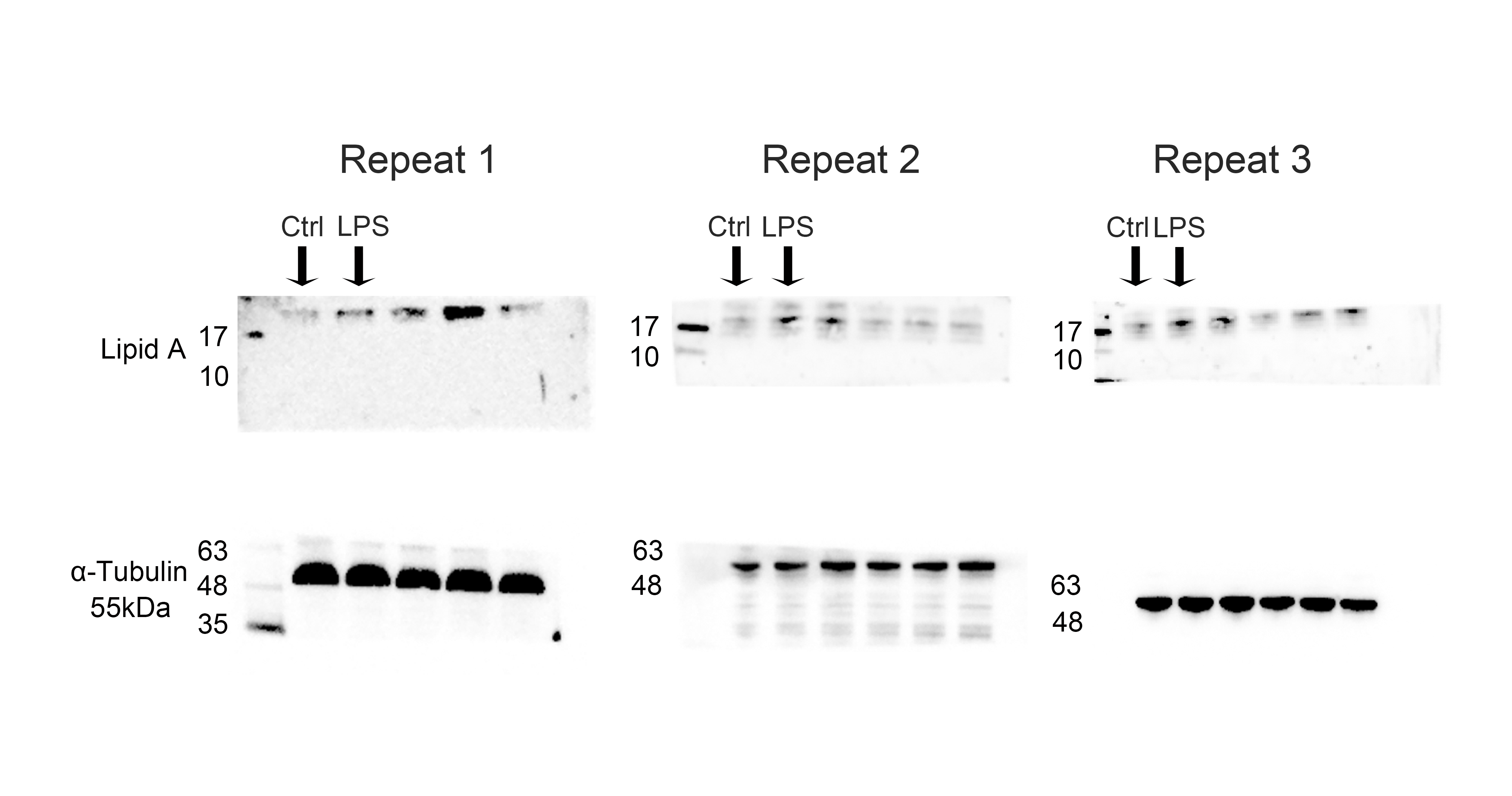

Supplement: Supplementary file 1 [file Data_Sheet_1.zip › Data sheet/Raw data of Western blot.tif]
