# Supplementary material for: A single dose of lipopolysaccharide elicits autofluorescence in the mouse brain
Source: Front Aging Neurosci. 2023 Mar 20;15:1126273. doi: 10.3389/fnagi.2023.1126273 (PMC10067636; doi:10.3389/fnagi.2023.1126273)
Supplement: Supplementary file 2 [file Presentation_1.PDF]

Supplementary Figures

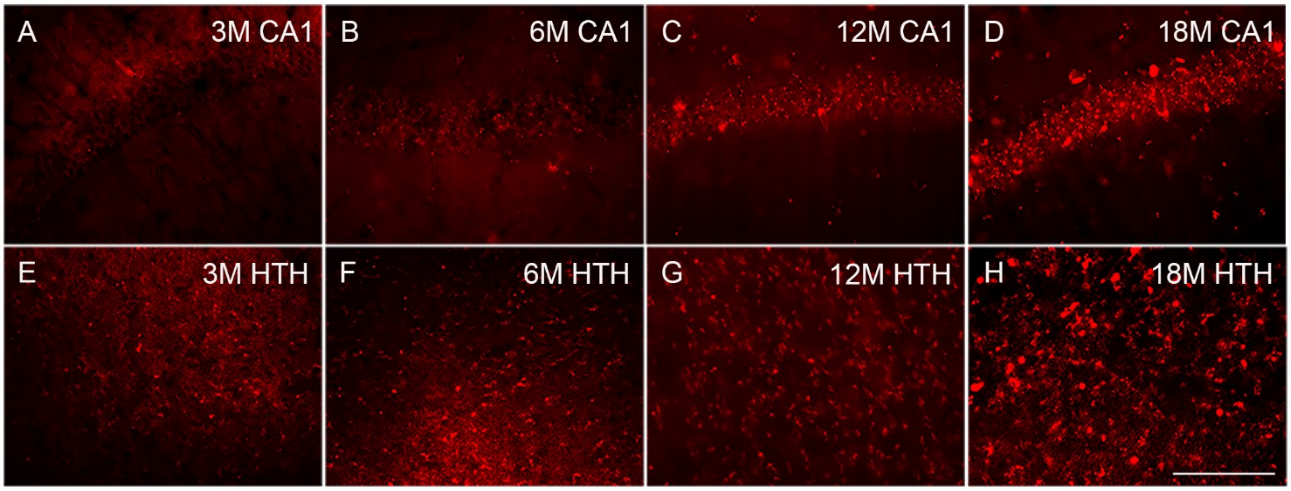

Supplementary Figure S1. The age-related AF in the hippocampus and hypothalamus**.** Expression of AF in the CA1 region of the hippocampus (CA1) from three (3M) to 18 months (18M) (**A-D**). Expression of AF in the hypothalamus (HTH) from three to 18 months (**E-H**). The scale bar represents 50 µm.

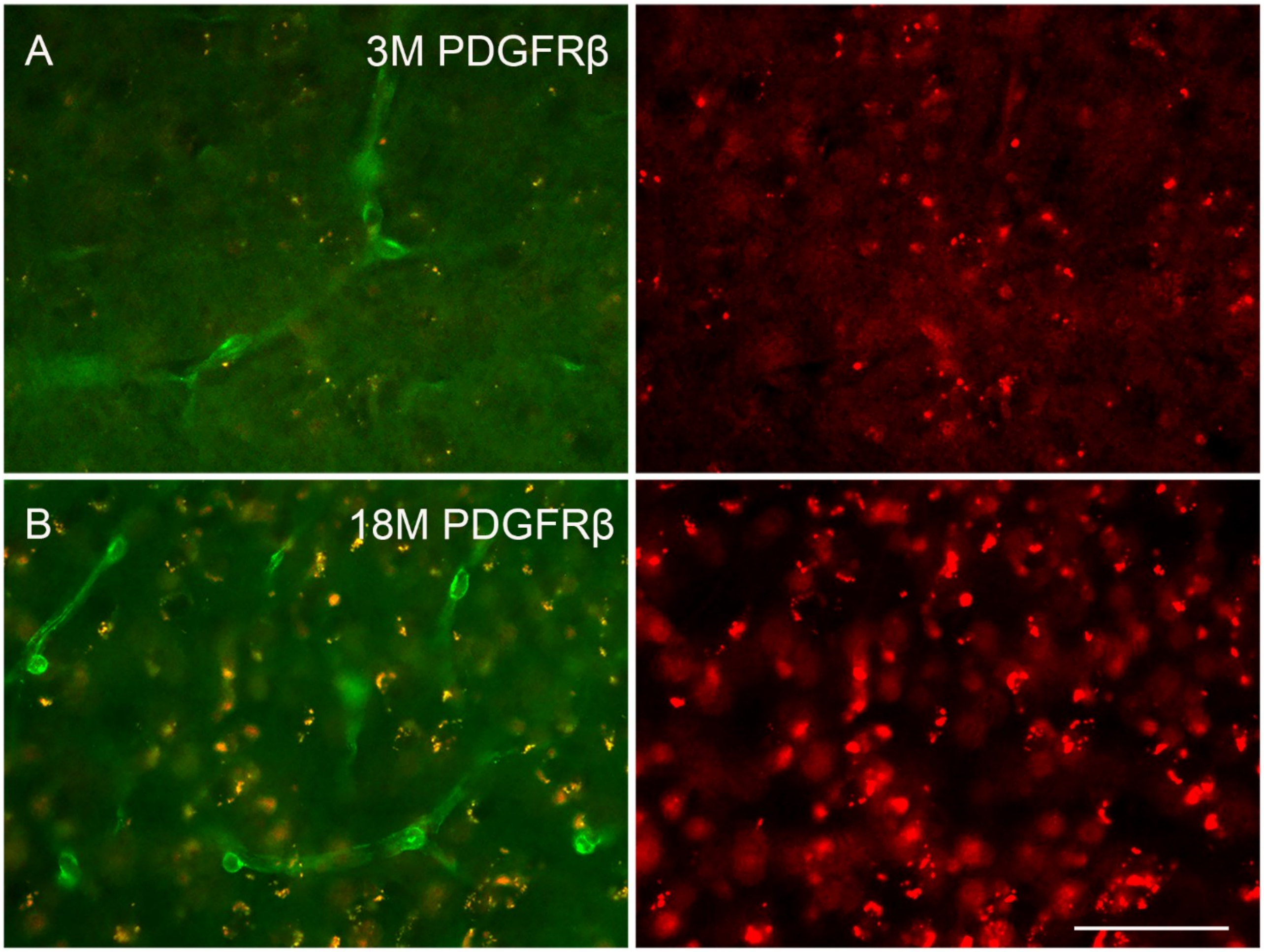

Supplementary Figure S2. AF expression in pericytes of young and aged mice. The representative immunofluorescence images of PDGFRβ. PDGFRβ staining was performed in three months (3M, **A**) and 18 months (18M, **B**) old mice and AF was seen in the rhodamine channel. The scale bar represents 20 μm.

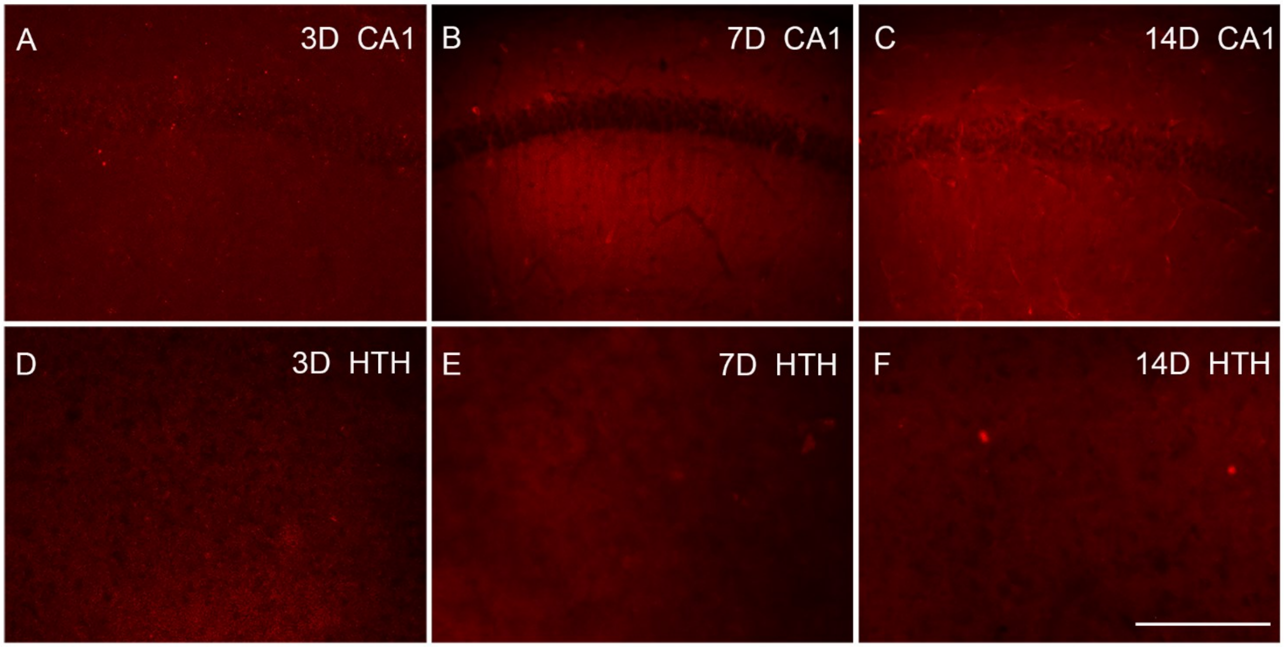

Supplementary Figure S3. Expression of AF induced by LPS injection inside the hippocampal CA1 region (CA1) and hypothalamus (HTH). AF inside the hippocampus and hypothalamus at three days (3D), seven days (7D), and 14 days (14D) after LPS injection**.** AF in the CA1 region of the hippocampus (CA1) at three days (3D, **A**), seven days (7D, **B**), and 14 days (14D, **C**) after LPS injection. AF within the hypothalamus (HTH) at three days (3D, **D**), seven days (7D, **E**), and 14 days (14D, **F**) post-LPS injection. The scale bar represents 50 µm.

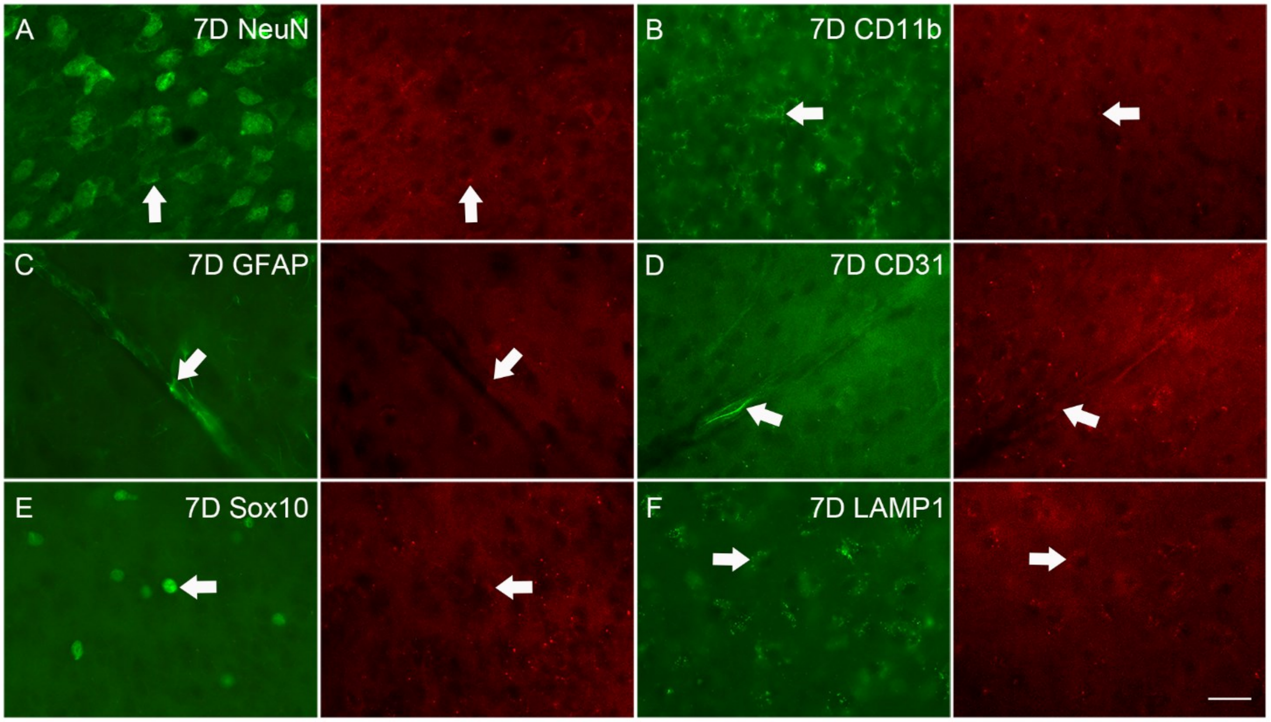

Supplementary Figure S4. The representative immunofluorescence images at seven days (7D) after LPS injection. The immunofluorescence and rhodamine channel imaging of NeuN (**A**), CD11b (**B**), GFAP (**C**), CD31 (**D**), Sox10 (**E**), LAMP1 (**F**). The arrows indicate fluorescence coincident with AF. The scale bar represents 20 μm.

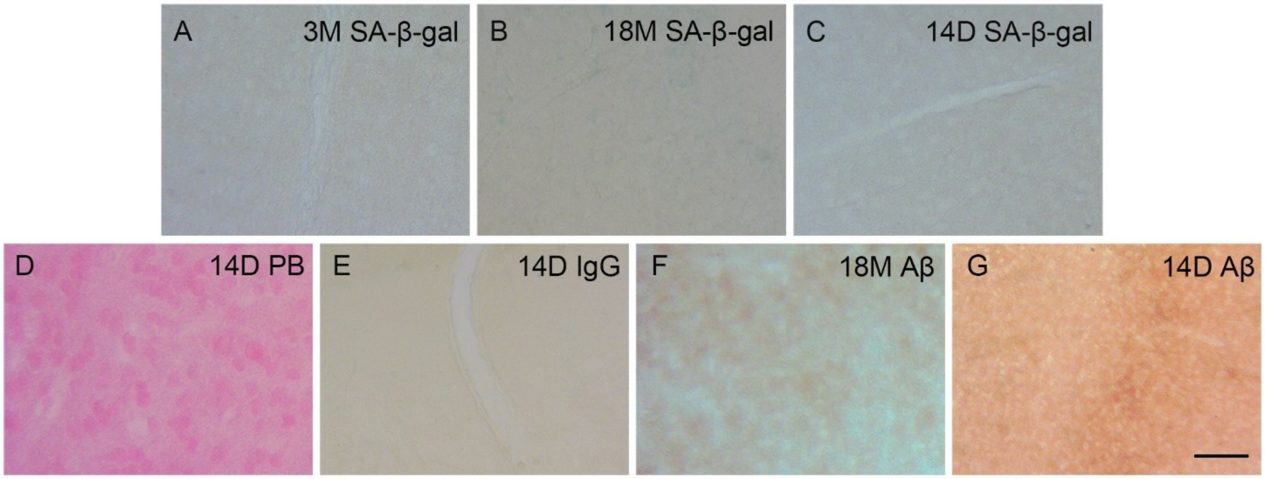

Supplementary Figure S5. The staining of aging and vascular leakage associated biomarkers in the mouse brain tissues. SA-β-Gal staining results of three-month (3M, **A**) and 18-month (18M, **B**) old mice. Mice 14 days (14D) after LPS injection were stained using SA-β-Gal (**C**), Prussian blue (**D**, counterstained with eosin), and IgG (**E**). Aβ42 staining results within the cortex of 18 months (18M) old mice (**F**) and LPS-injected young mice (**G**). The scale bar represents 100 μm.
